# A Pilot Study on the Effects of Nut Consumption on Cardiovascular Biomarkers

**DOI:** 10.7759/cureus.8798

**Published:** 2020-06-24

**Authors:** Jacob J Adashek, David Redding

**Affiliations:** 1 Internal Medicine, University of South Florida, Tampa, USA; 2 Neuromusculoskeletal Medicine/Osteopathic Manipulative Medicine/Family Medicine, Western University of Health Sciences, Pomona, USA

**Keywords:** lipid, cardiovascular health, atherosclerosis, almonds, hazelnuts, walnuts

## Abstract

Background: Cardiovascular disease (CVD) is a leading cause of morbidity and mortality in the United States and changes in lifestyle can minimize the likelihood of succumbing to heart disease. Anti-inflammatory agents are commonly used to reduce the chronic inflammatory state behind the pathogenesis of CVD. Multiple studies have been published correlating nut consumption with a reduction in both heart attacks and strokes. The goal of this study is to determine to what extent the consumption of almonds, hazelnuts, and walnuts have on the blood markers associated with cardiac disease and inflammation.

Methods: This was a six-week study in which subject’s baseline values act as controls. Fasting blood draws occurred at week 0, week 2, and after four weeks of intervention (week 6). All participants had undesirable lipid profiles and no medications related to heart disease.

Results: Total cholesterol (TC): high-density lipoprotein (HDL-C) ratio was lowered a statistically significant amount at the six-week time point (3.89 ± 0.74) compared to both the zero-week (4.93 ± 1.16, p < 0.01) and two-week (4.63 ± 1.20, p < 0.5) timepoints. Low-density lipoprotein (LDL) measurements were lowered a statistically significant amount at the six-week time point (135.6 ± 15.0 mg/dL) compared to the zero-week (159.7 ± 12.3 mg/dL, p < 0.01). Erythrocyte sedimentation rate (ESR) was lowered a statistically significant amount at six-week time point (10.44 ± 5.05 mm/h) compared to the zero-week (14.44 ± 5.12 mm/h, p < 0.01).

Conclusions: Blood markers associated with CVD specifically and the general marker for inflammation associated with many chronic diseases can be favorably modified with the consumption of specific nuts as demonstrated by this study.

## Introduction

Cardiovascular disease (CVD) is a leading cause of morbidity and mortality in the United States and worldwide [1]. The current recommendation by the American College of Cardiology is to encourage a cardioprotective diet in all individuals regardless of the disease state [2]. This recommendation was based on the following conclusion made by the 2015 Dietary Guidelines Advisory Committee [3] in its systematic review of the evidence on nut consumption and CVD risk: “There is moderate evidence that consumption of unsalted peanuts and tree nuts, specifically walnuts, almonds, and pistachios, in the context of a nutritionally adequate diet and when total calorie intake is held constant, has a favorable impact on cardiovascular disease risk factors, particularly serum lipid levels.” It is also recognized that inflammation plays a major role in all stages of the atherothrombotic process and that the consumption of nuts has a favorable influence on inflammatory markers [4].

Atherosclerosis, the most common cause of heart disease, has been widely linked to high cholesterol levels. However, it is now widely accepted that the innate immune system triggers cytokines and chronic inflammation. The key hallmark of an atheromata is lipid-laden “foam cells” that originate from mononuclear phagocytes [5]. Erythrocyte sedimentation rate (ESR) is a nonspecific biomarker of inflammation, but has been linked to influence blood viscosity and cardiovascular mortality [6].

After extensive literature review, it was found that there are no published studies using this combination of nuts to assess cardiovascular risk determining markers (CRDM) or inflammatory markers such as ESR. It was hypothesized that consuming the daily combination of nuts we prescribed would improve lipids, lipoproteins, and apolipoproteins and decrease ESR. Additionally, our study is unique in that we investigated the anti-inflammatory effects of the above-mentioned nuts by monitoring ESR levels.

## Materials and methods

Patients from the City of Montclair’s Medical Clinic (county of San Bernardino, CA) who were identified as having elevated cholesterol levels were invited in 2009 to participate in the study. This clinic is the city’s service to the local community that is supported by family medicine residents of the Montclair Doctors Hospital. Some 11 patients were enrolled in the study, which consisted of eight women and three men with an average age of 52. All patients were of Mexican descent and were not taking any medications or supplements (i.e. red yeast rice or fish oil) related to cholesterol. Patients were instructed to not make any changes to their diet or exercise programs, but to only introduce the consumption of specific nuts on a daily basis and surveyed weekly to assure compliance. 

The exclusion factors included having a known allergy to any nut or if the potential subject had any type of bleeding disorders (based on the recognition that each subject would be receiving three blood draws for the study and wanting to avoid any associated problems). Any previous history or current use of any lipid lowering agents (e.g. statins, fibrates, niacin, etc.) also excluded patient subjects.

The subjects acted as their own controls and all blood draws were collected after fasting. Laboratory markers included: total cholesterol (TC), triglycerides, low-density lipoprotein (LDL-C), high-density lipoprotein (HDL-C) levels, and ESR. The clinical endpoints were measured at three time points, specifically: the beginning of the study, at two weeks, and at six weeks after the nut consumption protocol was introduced into their diets beginning at week 2.

The protocol consisted of three almonds, three hazelnuts, and three walnuts to be consumed each morning for four weeks. To ensure that this happened, each subject received 28 packets, each filled with a day’s ration of nuts, one for each day of the study. This regimen of nuts and number of nuts was devised based on previous studies with each of the following nuts [7-9].

## Results

Following the six-week period of investigation, patients achieved notable changes in their serum lipids, lipoproteins, and apolipoproteins and ESR. Our data collection and analysis demonstrated several significant mean differences between our baseline, control, and protocol periods across all five parameters measured, namely TC, HDL-C, TC:HDL-C ratio, LDL-C, triglycerides, and ESR (Table 1). It is critical to note that two of the 11 patients lacked some portion of their laboratory values; the change in sample size will be noted with regard to each laboratory test as the results are reported.

**Table 1:** Serum lipid biomarkers demonstrating the effect of nut consumption protocol. Serum lipid biomarkers demonstrating the effect of nut consumption protocol; p-values represent general differences between our baseline, control, and nut-enriched diet periods (p < 0.05; significantly different). Values are means ± standard deviations. HDL, high density lipoprotein; TC, total cholesterol; LDL, low density lipoprotein; ESR, erythrocyte sedimentation rate

Variables	Sample size (n)	Baseline (t = 0)	Diet periods		p-value
			Control (t = 2)	Nut-enriched (t = 6)	
		mg/dL			
Total cholesterol	11	251.0 ± 24.8	246.6 ± 24.3	226.1 ± 25.6	0.017930
HDL cholesterol	10	53.1 ± 13.7	55.4 ± 13.6	61.5 ± 13.2	<0.0001
TC:HDL-C ratio	10	4.93 ± 1.16	4.63 ± 1.20	3.89 ± 0.74	0.000630
LDL cholesterol	11	159.7 ± 12.3	153.6 ± 10.1	135.6 ± 5.0	<0.000119
Triglycerides	10	186.4 ± 38.0	155.1 ± 30.7	129.3 ± 37.4	<0.0001
		mm/h			
ESR	9	14.4 ± 5.1	14.3 ± 6.3	10.4 ± 5.1	0.00011

Total cholesterol was reduced in eight of the 11 patients and there was a statistically significant difference between measurement points as determined by one-way analysis of variance (ANOVA) for correlated samples [F(2,20) = 4.95, p = 0.017930]. A Tukey post-hoc test revealed that the TC was lowered a statistically significant amount at six-week time point (226.1 ± 25.6 mg/dL, p < 0.05) compared to the zero-week (251.0 ± 24.8 mg/dL,) time point. There were no significant differences noted between the TC measurements at the zero-week and two-week time points (246.6 ± 24.3, p = 0.859), or were there significant differences noted between the two-week and six-week time points (p = 0.0620).

HDL-C was increased in 10 patients with a complete post-protocol record and there was a statistically significant difference between measurement points as determined by one-way ANOVA for correlated samples [F(2,18) = 28.79, p < 0.0001]. A Tukey post-hoc test revealed that the HDL measurements were raised a statistically significant amount at six-week time point (61.5 ± 13.2 mg/dL) compared to both the zero-week (53.1 ± 13.7 mg/dL, p < 0.01) and two-week (55.4 ± 13.6, p < 0.01) time points respectively. There were no significant differences noted between the HDL measurements at the zero-week and two-week time points (p = 0.138).

The TC:HDL-C ratio was decreased in 10 patients with a complete post-protocol record and there was a statistically significant difference between measurement points as determined by one-way ANOVA for correlated samples [F(2,18) = 11.41, p = 0.000630]. A Tukey post-hoc test revealed that TC:HDL-C ratio was lowered a statistically significant amount at the six-week time point (3.89 ± 0.74) compared to both the zero-week (4.93 ± 1.16, p < 0.01) and two-week (4.63 ± 1.20, p < 0.5) time points respectively. There were no significant differences of the TC:HDL-C ratio noted between the zero-week and two-week time points (p = 0.3984).

LDL-C levels were reduced in all 11 patients and there was a statistically significant difference between the measurement points as determined by one-way ANOVA for correlated samples [F(2,20) = 14.69, p < 0.000119]. A Tukey post-hoc test revealed that the LDL measurements were lowered a statistically significant amount at six-week time point (135.6 ± 15.0 mg/dL) compared to both the zero-week (159.7 ± 12.3 mg/dL, p < 0.01) and two-week (153.6 ± 10.1, p < 0.01) time points respectively. There were no significant differences noted between the LDL measurements at the zero-week and two-week time points (p = 0.402).

Triglyceride levels were found to be reduced at all post-protocol measurement points across 10 patients with a complete data set and there was a statistically significant difference between measurement points as determined by one-way ANOVA for correlated samples [F(2,18) = 18.74, p < 0.0001]. A Tukey post-hoc test revealed that the triglyceride measurements were lowered a statistically significant amount at six-week time point (129.3 ± 37.4 mg/dL) compared to both the zero-week (186.4 ± 38.0 mg/dL, p < 0.01) and two-week (155.1 ± 30.7, p < 0.05) time points respectively. There was also a statistically significant difference between the measurements at the zero-week and two-week time points (p < 0.01).

The ESR was decreased at all post-protocol measurement points across nine patients with complete datasets and there was a statistically significant difference between measurement points as determined by one-way ANOVA for correlated samples [F(2,16) = 17.01, p = 0.00011]. A Tukey post-hoc test revealed that the ESR was lowered a statistically significant amount at six-week time point (10.44 ± 5.05 mm/h) compared to both the zero-week (14.44 ± 5.12 mm/h, p < 0.01) and two-week (14.33 ± 6.25, p < 0.01) time points respectively. There were no significant differences noted between the ESR measurements at the zero-week and two-week time points (p = 0.989).

## Discussion

The purpose of this research was twofold; determining to what extent the consumption of specific nuts would have on the blood markers associated with CVD and evaluating if nut consumption reduced the level of inflammation. This study answers both questions.

The ability of almonds to lower LDL-C levels in a dose-dependent fashion has been well documented [10-11]. Hazelnuts have been noted for their unique fatty acid composition specifically high monounsaturated fatty acid contents, which are demonstrated to lower TC levels and LDL-C levels [12]. Lastly, walnuts are shown to decrease LDL-C levels and diastolic blood pressure readings [13]. Although the American College of Cardiology also specifically mentions that peanuts and pistachios reduce serum lipid levels, they were not included in our study.

**Figure 1:**
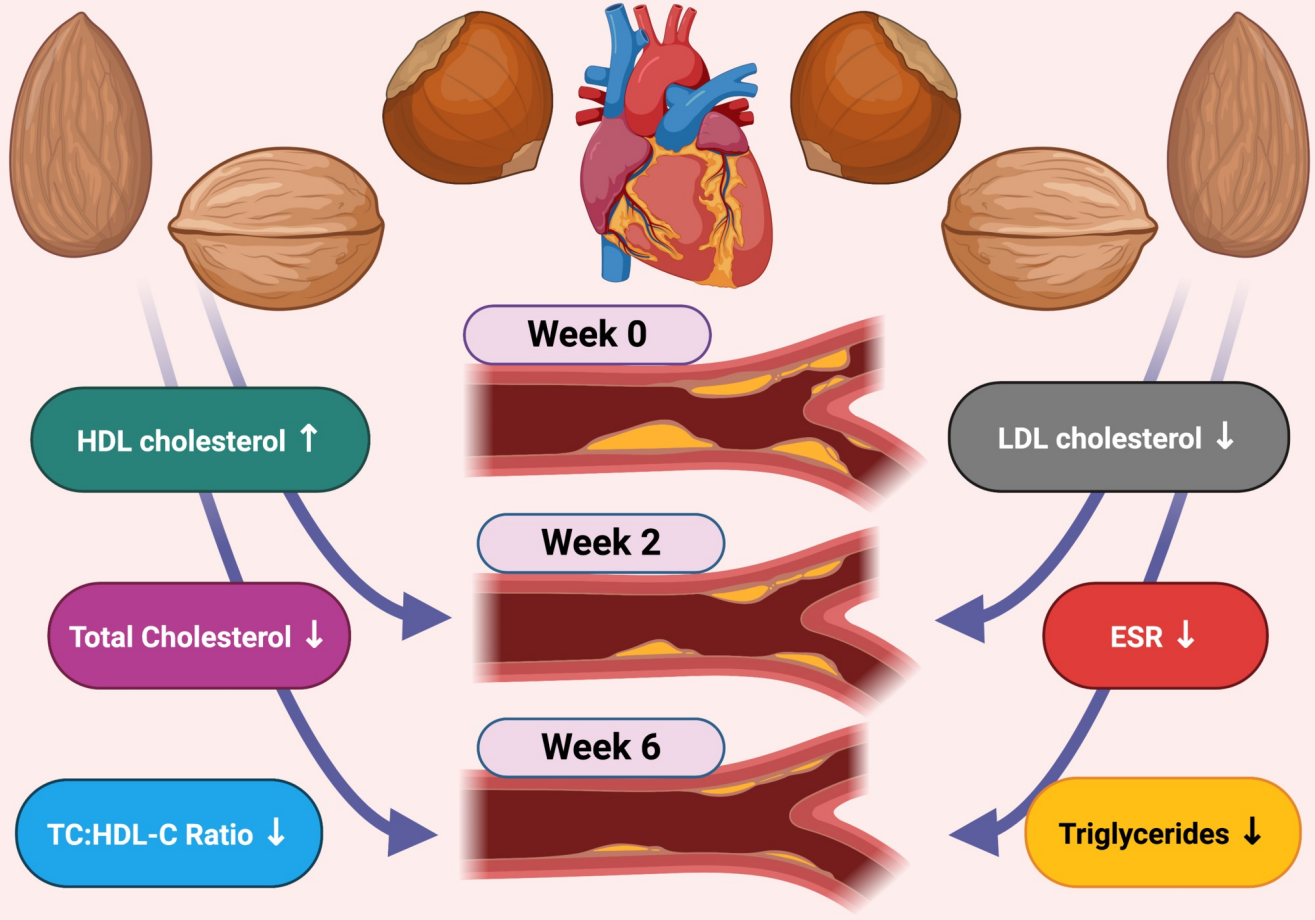
Effects on cardiovascular biomarkers.

This combination of nuts is unique and unequivocally demonstrates that regimented consumption of a modest amount of almonds, hazelnuts, and walnuts can, over a four-week interval, manifest significant changes in serum measurements of LDL-C, HDL-C, triglycerides, and ESR (Figure 1).

These findings point to the critical role that dietary modification and lifestyle interventions have in implementing a comprehensive management plan for patients with an increased risk for developing CVD.

The most promising of our results is the reduction in TC:HDL-C ratio, which was statistically significant. The TC:HDL-C ratio is considered one of the most predictive laboratory measurements to best predict coronary atheroma progression and cardiovascular-related events [14].

The statistically significant decrease in triglycerides between week 0 and week 2 was noted, but as there was no intervention administered between this time, the authors do not have an explanation for this. Perhaps, despite being told not to change dietary habits, Hawthorne effect took place and some of the subjects made changes to their diets.

Some of the limitations of this study include the paucity of subjects, fund availability, and number of markers followed. The authors also note a lack of generalizability as the study predominantly evaluated those of Hispanic descent, the majority of whom were women, but still believe that the data and findings are valuable for a pilot study. Additionally, the principal investigator of the study had intended to use ESR and high sensitivity C-reactive protein (hs)CRP as a level, but due to issues with the laboratory collecting and reporting only ESR was included in the study. This is the same issue that caused two of the data points to not be reported and the data reflect this. Future directions for expanding this line of inquiry include recruiting a larger cohort, expanding the laboratory panel to further track changes in systemic inflammatory burden, a longer longitudinal outcome measurement, and, ultimately, correlating patient outcomes with the nut consumption protocol.

## Conclusions

This study reaffirms the important impact that a healthy diet, specifically nuts, can have on both the preventative and management aspects of CVD. The daily consumption of these nuts produced a favorable change on the cardiovascular risk markers and especially important is the TC:HDL-C ratio and inflammation in general. There are no other studies in the current literature that link the consumption of nuts with the reduction of inflammation by measuring ESR, which we found to be statistically significant despite of the small number of subjects and the relatively small number of nuts consumed daily. Our data suggest that nut consumption reduces inflammation; the vast majority of chronic diseases are associated with higher inflammatory states. Nut consumption is protective and explains why nut consumers have a “reduction in all-cause mortality and morbidity.” A meta-analysis with over 6300 patients showed that one serving of unspecified nut consumption per week decreased coronary heart disease risk. Our data suggest that a daily intake of specific nuts may have even a greater positive impact on heart disease and it is our hope that nut consumption will become a recognized standardized approach in all patients with cardiac disease.

Further studies could assess the molecular mechanisms that nut consumption has on cholesterol metabolism and inflammation. The recommendation to consume nuts as part of daily diet has shown in many studies and in our study to be cardioprotective and anti-inflammatory. Discovering the mechanisms of how lipid and inflammation levels are decreased could provide more insight into our nut protocol as well as how effective other combinations of nuts are in decreasing these levels.
